# Human sex reversal is caused by duplication or deletion of core enhancers upstream of *SOX9*

**DOI:** 10.1038/s41467-018-07784-9

**Published:** 2018-12-14

**Authors:** Brittany Croft, Thomas Ohnesorg, Jacqueline Hewitt, Josephine Bowles, Alexander Quinn, Jacqueline Tan, Vincent Corbin, Emanuele Pelosi, Jocelyn van den Bergen, Rajini Sreenivasan, Ingrid Knarston, Gorjana Robevska, Dung Chi Vu, John Hutson, Vincent Harley, Katie Ayers, Peter Koopman, Andrew Sinclair

**Affiliations:** 1 Murdoch Children’s Research Institute, Melbourne, 3052 VIC Australia; 2 Department of Molecular & Translational Science, Monash University, Clayton, 3800 VIC Australia; 3 Department of Paediatrics, The University of Melbourne, Melbourne, 3010 VIC Australia; 4 School of Bioscience, University of Melbourne, Melbourne, 3010 VIC Australia; 5 Department of Paediatric Urology, Monash Children’s Hospital, Clayton, 3168 VIC Australia; 6 School of Biomedical Sciences, University of Queensland, Brisbane, 4072 QLD Australia; 7 Institute for Molecular Bioscience, The University of Queensland, Brisbane, 4072 QLD Australia; 8 Bioinformatics Division, Walter & Eliza Hall Institute of Medical Research, Melbourne, 3052 VIC Australia; 9 Hudson Institute for Medical Research, Clayton, 3168 VIC Australia; 10 Department of Medical Genetics, Metabolism & Endocrinology, National Children’s Hospital, Hanoi, Vietnam; 11 Department of Urology, Royal Children’s Hospital, Melbourne, 3052 VIC Australia

**Keywords:** Gene expression, Gene regulation

## Abstract

Disorders of sex development (DSDs) are conditions affecting development of the gonads or genitalia. Variants in two key genes, *SRY* and its target *SOX9*, are an established cause of 46,XY DSD, but the genetic basis of many DSDs remains unknown. SRY-mediated *SOX9* upregulation in the early gonad is crucial for testis development, yet the regulatory elements underlying this have not been identified in humans. Here, we identified four DSD patients with overlapping duplications or deletions upstream of *SOX9*. Bioinformatic analysis identified three putative enhancers for *SOX9* that responded to different combinations of testis-specific regulators. All three enhancers showed synergistic activity and together drive *SOX9* in the testis. This is the first study to identify *SOX9* enhancers that, when duplicated or deleted, result in 46,XX or 46,XY sex reversal, respectively. These enhancers provide a hitherto missing link by which SRY activates *SOX9* in humans, and establish *SOX9* enhancer mutations as a significant cause of DSD.

## Abstract (web summary)

SRY and its target SOX9 are known key determinants in testis development. Here the authors by studying duplications and deletions upstream of SOX9 from patient samples with disorders of sex development (DSD) reveal enhancers for SOX9 critical for human sex development and DSD.

## Introduction

Gonadal sex differentiation begins in the embryo with the development of the bipotential gonads into either testes or ovaries. In most mammals including humans, testis determination is initiated by the Y-linked gene *SRY*^1,2^. *Sry* is expressed transiently from 10.5 days post coitum (dpc) in mouse embryos^3–5^ while in humans expression begins at 6 weeks post ovulation and is maintained throughout gestation^6^. SRY acts by upregulating the gene encoding the transcription factor SOX9, which is both necessary and sufficient for testis development and hence male differentiation in mice and humans^7–9^. How SRY activates *SOX9* transcription is a major unresolved issue. *SOX9* tissue specific expression is influenced by long-range regulatory elements (including enhancers, which are orientation-independent controllers of expression) within  the 2 Mb upstream of the transcriptional start site^10^. In mice, it is known that SRY co-operates with NR5A1 (SF1) to bind to and activate a *Sox9* enhancer known as Testis Specific Enhancer of Sox9 (TES) and its core sequence known as TESCO^11,12^. Once *Sox9* expression begins, SOX9 auto-regulates its own transcription via TESCO, creating a positive feedback loop^11^. However, loss of function of TESCO in mice results in reduced *Sox9* expression but this is insufficient to cause sex reversal^12^. This indicates that TESCO is not the only enhancer required for *Sox9* expression. In light of this, a recent study has identified a new mouse long-range enhancer for *Sox9* gonadal expression (denoted Enh13). When this enhancer is deleted, *Sox9* expression is markedly reduced resulting in complete XY sex reversal^13^.

In recent years, several isolated 46,XX and 46,XY DSD patients with copy number variants (CNVs) within the 2 Mb putative *SOX9* upstream regulatory region have been identified, denoted XYSR and RevSex (reviewed by Croft et al.^14^). In addition, despite extensive research, no DSD patients with pathogenic variants in TESCO have been identified^15^. Thus, while studies have defined two enhancers necessary for *Sox9* regulation in the mouse, to date, the testis-specific enhancers have yet to be identified in humans.

In this study we redefine the upstream regulatory landscape of human *SOX9*. Using new patient data, we refined the 32.5 kb XYSR and 24 kb RevSex intervals and analysed the genomic regions using bioinformatic and luciferase tiling approaches, to identify three putative enhancers 5′ of *SOX9*. In cell-based reporter assays these enhancers responded to different combinations of testis-specific regulators including SRY, SF1 and SOX9 itself. When combined, all three enhancers show synergistic activity, significantly increasing their individual enhancer activity. In vivo, deletion of these three enhancers in mice resulted in different outcomes ranging from: no apparent effect to reduced *Sox9* transcription and complete sex reversal. Given that duplication or deletion of these sequences results in sex reversal in *SRY*-negative 46,XX males and 46,XY females, respectively, our results suggest a mechanism by which these enhancers have crucial roles in human sex development and DSD.

## Results

### A human *SOX9* enhancer associated with 46,XX and 46XY DSD

**Fig. 1:**
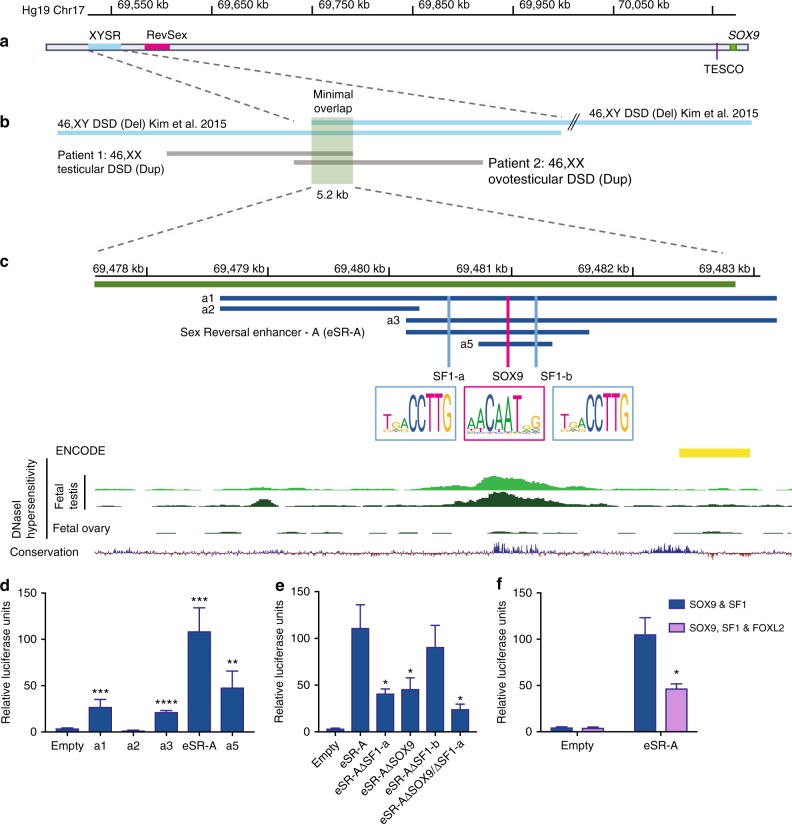
Duplication or deletion of the human *SOX9* testicular enhancer eSR-A is associated with DSD. **a** The 600 kb genomic region upstream of human *SOX9* showing the XYSR, RevSex and TESCO candidate regulatory regions. **b** The XYSR region was defined by deletions in two previously published 46,XY DSD patients (blue)^17^. Two novel duplications in 46,XX DSD patients (grey), allowed us to redefine the minimal overlap to 5.2 kb (green). **c** Sub-cloning strategy, dark blue lines indicate sub-clones analysed for enhancer activity in the pGL4.10 Beta-globin (βg) plasmid using luciferase assays. Predicted transcription factor binding motifs for SF1 (SF1-a and SF1-b) and SOX9 are shown with vertical light blue and magenta lines. Bioinformatic tracks from the UCSC genome browser are shown including the ENCODE track of enhancers present in human mammary epithelial cells (HMEC) (yellow denotes a weak enhancer)^28^ and DNaseI hypersensitivity data from human foetal testis and ovary (ROADMap)^23^. This shows a testis-specific peak over the a4 fragment. The 100-vertebrate conservation track shows a spike of conservation beneath the a4 fragment DNaseI peak. **d** Enhancer activities of sub-cloned fragments a1-a5 as measured by luciferase assays transfected with SF1 and SOX9 (*n* = 4). **e** Mutation of the SF1-a (eSR-A ΔSF1-a), and SOX9 (eSR-A ΔSOX9) binding motifs either separately or together results in a loss of enhancer activity compared to non-mutated eSR-A as assessed by luciferase assays with co-transfection of SF1 and SOX9 (*n* = 4). **f**. Co-transfection of FOXL2 with SF1 + SOX9 shows a repression of eSR-A activity compared to SF1 + SOX9 (*n* = 3). All luciferase assays carried out in COS7 cells. Error bars are s.e.m. *P*-values (two-tailed *t* tests): *P ≤ 0.05. **P ≤ 0.01, ***P ≤ 0.001, ****P ≤ 0.0001. Source data are provided as a Source Data file.

We focused on genetic intervals in the 2 Mb *SOX9* upstream regulatory region that were previously associated with 46,XY and 46,XX DSD^16–22^. First, we analysed a 32.5 kb region, known as XYSR (17q24.3), ~500 kb upstream of *SOX9* and TESCO, deletion of which causes 46,XY sex reversal^17^ (Fig. 1a). From a cohort of 44 DSD patients we identified two further duplications mapping to 17q24.3 in two unrelated 46,XX DSD patients lacking *SRY*. Patient 1 (46,XX testicular DSD) carries a 23.9 kb duplication, while Patient 2 (46,XX ovotesticular DSD) carries a 24.2 kb duplication (Fig. 1b and Supplementary Figure 1). CGH-array showed these duplications overlapped with each other and the XYSR region, defining a minimum critical region of 5.2 kb (Fig. 1b). Therefore, we anticipated that this redefined region would include a core gonadal enhancer for *SOX9* implicated in both 46,XY and 46,XX DSD (Fig. 1b).

Within the 5.2 kb minimal region, DNaseI hypersensitivity data revealed strong regulatory potential in human embryonic testes but not ovaries^23^ (Fig. 1c). The 5.2 kb minimum critical region was sub-cloned into five overlapping fragments (Fig. 1c). Each was tested for enhancer activity in vitro using luciferase reporter transcriptional assays together with testicular transcription factors known to regulate *SOX9*, including SRY, SF1 and SOX9 itself. Enhancer activity was tested in a variety of cell lines (COS7, TM3, TM4 and HEK293T) which all showed similar results (Supplementary Figure 2). Here, we present data derived from COS7 cells. Four of the five fragments showed significant enhancer activity when co-transfected with SF1 and SOX9 (Fig. 1d). The strongest regulatory response was observed with a 1514 bp fragment, that likely contains the core enhancer. We have named this genomic region, which showed a 100-fold increase in activity in the presence of SF1 and SOX9, Sex Reversal Enhancer-A [eSR-A] (Fig. 1c,d). This fragment contains one SRY/SOX9 and two SF1 consensus binding sites (Fig. 1c). Mutation of the SOX9 and one SF1 binding motif (SF1-a) separately or together caused a significant decrease in enhancer activity (78%); mutation of the second SF1 binding motif (SF1-b) had no effect, suggesting it is not functional (Fig. 1e). Co-expression of the ovarian transcription factor FOXL2, which antagonises *SOX9* activity in vivo^24,25^ resulted in a significant repression of enhancer activity (50%) (Fig. 1f). These data indicate that fragment eSR-A contains a *SOX9* enhancer that responds positively to testicular transcription factors in humans.

In a recent independent but complementary study on *Sox9* regulation in the mouse by Gonen and colleagues, an orthologous mouse enhancer (Enh13) showing 80% sequence conservation with eSR-A was identified^13^. When deleted, complete sex reversal in XY mice was observed^13^. In our system, mouse eSR-A (Enh13) demonstrated strong enhancer activity in response to SF1 in combination with mouse SOX9, and to SF1 in combination with mouse SRY. By contrast, the human eSR-A enhancer shows robust activation with SOX9 and SF1, but no activation with human SRY and SF1 (Supplementary Figure 3a,b). Thus, the mechanisms required to activate this enhancer may differ between species. Regardless, our data identifies human eSR-A as a critical, conserved *SOX9* testis-specific enhancer that when deleted results in 46,XY sex reversal and when duplicated causes 46,XX (ovo) testicular DSD in humans.

### A human *SOX9* enhancer contributes to *SOX9* autoregulation

**Fig. 2:**
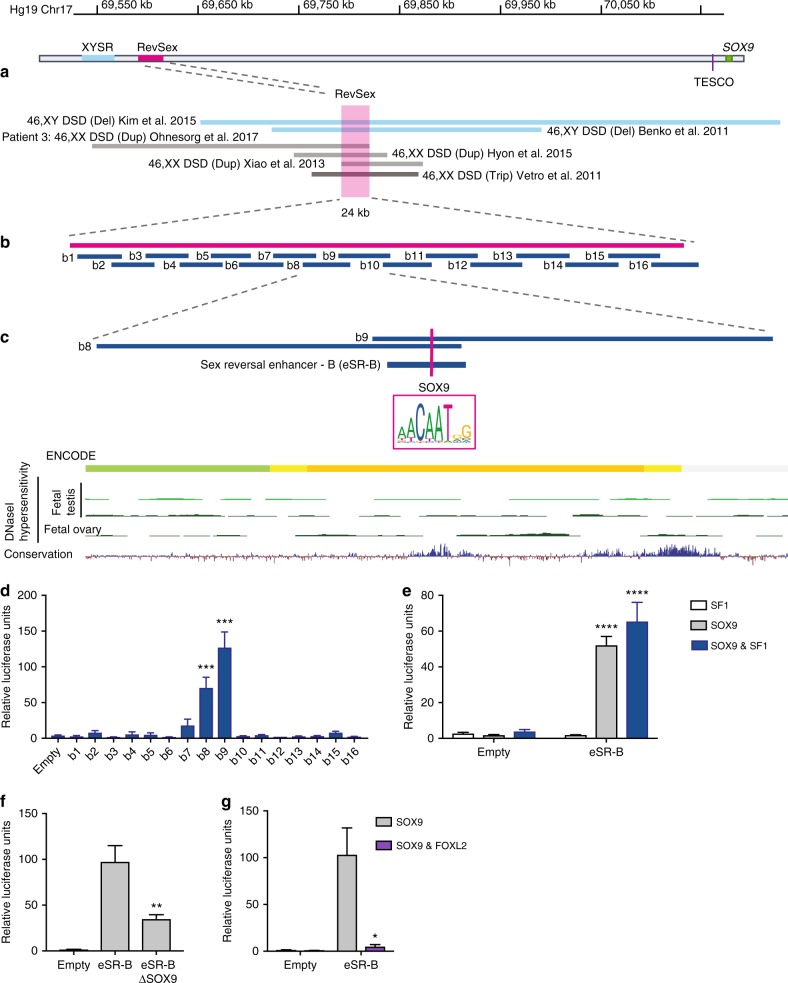
Identification and analysis of the SOX9 enhancer eSR-B. **a** Schematic of a number of previously published 46,XX DSD duplication/triplication (grey)^16,18,20,21^ and 46,XY DSD deletion (blue)^17,22^ patients upstream of *SOX9*. The minimal overlap (RevSex) of the six patients (24 kb) in magenta. **b** Unbiased luciferase tiling strategy of the minimal RevSex region (16 constructs—blue) to test for enhancer activity using the PGL4-βg vector. **c** Two fragments, b8 and b9, showed significant enhancer activity and their overlapping fragment, eSR-B was sub-cloned (blue lines). A conserved SOX9 binding site was identified (magenta line and box) and UCSC genome browser ENCODE data (HMEC) highlighted a strong active enhancer site (orange), conservation tract indicated a conserved sequence (blue peaks). No DNaseI peaks were detected in human foetal testis or ovaries (green). **d** Enhancer activities of small fragments (b1-b16) of the minimal overlap in COS7 cells transfected with SF1 and SOX9 in luciferase assays. Only the fragments b8 and b9 showed significant enhancer activity compared to the empty vector PGL4-βg (*n* = 3). **e** Enhancer activities of b8 and b9 overlapping region eSR-B as assessed by luciferase assays in COS7 cells co-transfected with SF1 (white bars) and SOX9 alone (grey bars) and in combination (blue bars) (*n* = 4). Significance is compared to the PGL4-βg empty vector control. **f** Enhancer activity of eSR-B fragment with mutation of the SOX9 (eSR-B ΔSOX9) binding site motif in response to SOX9 transfection (*n* = 7) significance compared to the eSR-B non-mutated fragment. **g** Luciferase reporter assays show FOXL2 transfected with SOX9 represses eSR-B enhancer activity compared to SOX9 alone (*n* = 4). All Luciferase assays are carried out in COS7 cells. Error bars are s.e.m. *P*-values derived from two-tailed *t* tests: *P ≤ 0.05, **P ≤ 0.01, ***P ≤ 0.001, ****P ≤ 0.0001. Source data are provided as a Source Data file.

Next, we focused on a previously identified upstream regulatory region of *SOX9*, RevSex^22^ (Fig. 2a). When the RevSex region was first described its minimal length was 178 kb^26^. The analysis of subsequent DSD patients carrying informative CNVs has allowed the minimal critical region to be narrowed to 41 kb^18^. This 41 kb region contains a strong enhancer signature previously confirmed by chromatin immunoprecipitation followed by sequencing (ChIP-seq)^27^. Recently, we further refined the minimal RevSex region to 24 kb by analysing a duplication in a 46,XX ovotesticular DSD patient (patient 3) overlapping those previously described^16^ (Fig. 2a).

To locate the enhancer element within RevSex we employed an unbiased screen, generating 16 overlapping ~2 kb fragments covering this 24 kb region (Fig. 2b) and testing each for enhancer activity using luciferase assays as described above (Fig. 2d–f). Two fragments (b8 and b9) showed a significant increase in enhancer activity in response to SF1 and SOX9 (Fig. 2c,d). These two fragments have a 416 bp overlap that contains a highly conserved SOX9 binding motif. ENCODE data show a binding site for p300 (a general transcription factor often associated with enhancers) and a DNaseI hypersensitive sites highlighting the regulatory potential of this genomic region^27,28^ (Fig. 2c). The 416 bp fragment also showed significant reporter activity when transfected constructs expressing SOX9 alone (Fig. 2e), and mutation of a SOX9 binding motif led to a 60% reduction in reporter expression levels (*P* = 0.0053, *t*-test) (Fig. 2f). We designated this fragment Sex Reversal Enhancer-B (eSR-B).

In addition, FOXL2 almost abolished eSR-B enhancer activity despite the stimulatory effect of SOX9 (Fig. 2g). Human eSR-B reporter transgenic mice showed equivalent expression in both testis and ovaries at E12.5 however, by E13.5 and E14.5 human eSR-B levels decreased in male gonads (Supplementary Figure 4a–c). Immunofluorescence on sections of E12.5 XY gonads showed that transgene expression was restricted to the testis cords, in particular in the Sertoli cells (indicated by co-staining with the Sertoli marker SOX9) (Supplementary Figure 4h & i). In the ovary at E12.5 and E13.5, the reporter was co-expressed with the granulosa cell marker FOXL2 (Supplementary Figure 4d–g). This suggests that the human eSR-B is responsive to factors in the gonads from E12.5 onwards, consistent with response to SF1 + SOX9 and SOX9 alone in vitro. eSR-B shares 75% sequence conservation in mouse. Like human eSR-B, mouse eSR-B demonstrated activation by mouse SOX9 in in vitro luciferase assays, albeit to a lesser extent (Supplementary Figure 5a, b). We therefore generated a CRISPR/Cas9 deletion of the mouse eSR-B region. These mice showed no obvious gonadal or sex reversal phenotype at either embryonic or adult stages (Supplementary Figure 6a–p) and no significant changes in *Sox9*, *Wnt4*, *Foxl2* or *Amh* mRNA expression levels (Supplementary Figure 6q-t). This suggests that eSR-B is a human-specific *SOX9* gonadal enhancer that when disrupted can cause sex reversal.

### Human *SOX9* testis enhancer eALDI is activated by SRY and SF1

**Fig. 3:**
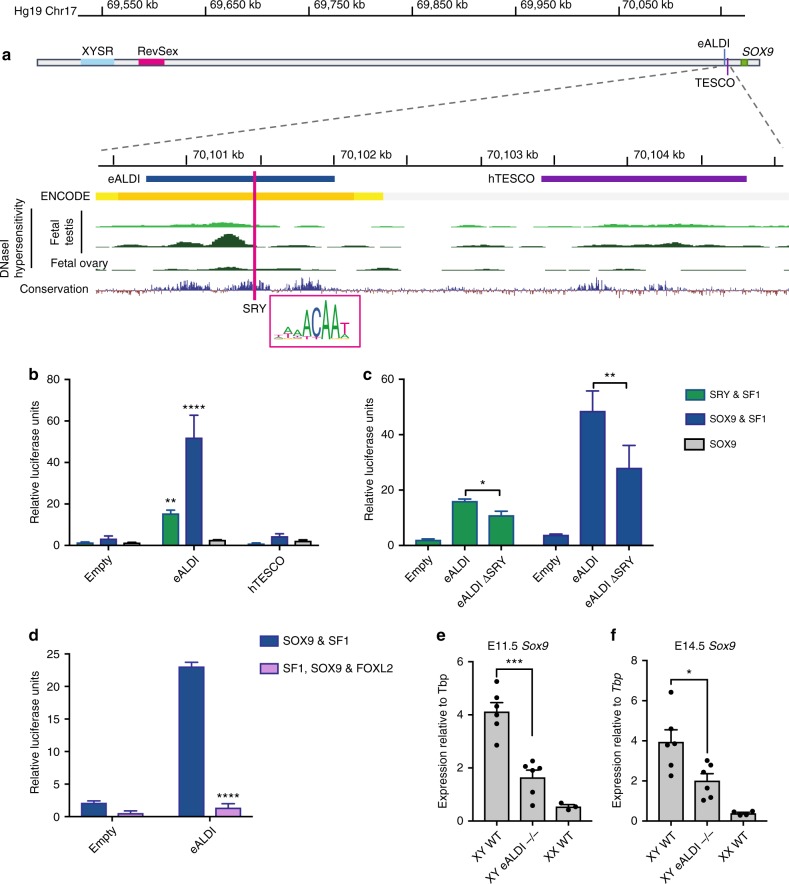
Identification and analysis of the SOX9 enhancer eALDI. **a** Schematic of the upstream region of *SOX9* highlighting human TESCO (hTESCO) and the proposed enhancer eALDI. Bioinformatic data from UCSC genome browser. ENCODE data (HMEC) indicated a strong active enhancer (orange) covering the proposed eALDI region. DNaseI peaks were observed in one foetal testis track (dark green), and strong conservation across the region was observed (blue peaks). A predicted SRY transcription factor binding motif is presented by magenta vertical line and box. By comparison ENCODE and DNaseI data for hTESCO show less enhancer potential and lower levels of conservation. **b** Enhancer activity of eALDI and hTESCO measured by luciferase assays in COS7 cells when co-transfected with SOX9 alone (grey bars), SOX9 + SF1 (blue bars) and SF1 + SRY (green bars). The eALDI region shows significant enhancer activity in response to both SF1 + SOX9 and SF1 + SRY transfection when compared to the empty pgl4-βg (*n* = 4). **c** Mutation of the SRY (eALDIΔSRY) transcription factor motif in the eALDI enhancer reduced activity when co-transfected with SF1 + SOX9 or SF1 + SRY compared to unmuted eALDI. Error bars represent s.e.m. *P*-values derived from two-tailed *t* tests: **P ≤ 0.01, ****P ≤ 0.0001. **d** Luciferase reporter assays in COS7 show FOXL2 transfected with SF1 + SOX9 represses eALDI enhancer activity compared to co-transfection with SOX9 + SF1 alone (*n* = 3) Error bars are s.e.m. *P*-values derived from two-tailed *t* tests: ****P ≤ 0.0001. **e** CRISPR/Cas9 mediated knock-out of eALDI resulted in reduction of *Sox9* expression to 40% of wild type at E11.5 and **f** 51% of wild-type levels in mouse testis at E14.5 as assessed by qRT-PCR. At 11.5 *n* = 6 XY WT, XY eALDI *n* = 6 and XX WT *n* = 3. At E14.5 XY WT *n* = 6, XY eALDI *n* = 6 and XX WT *n* = 4. Error bars are s.e.m. *P*-values derived from *t-*tests: *P ≤ 0.05 and ***P < 0.001 compared to wild-type males. Source data are provided as a Source Data file.

As both eSR-A and eSR-B enhancers are duplicated in *SRY*-negative 46,XX DSD patients, they can be activated without SRY. Indeed, our in vitro data have shown that eSR-A and eSR-B have a greater response to SOX9 + SF1 than to SRY + SF1. Therefore, in an effort to locate an SRY-responsive enhancer of *SOX9* in humans we performed a bioinformatics screen, looking for conserved enhancer marks upstream of *SOX9* from publicly available databases, with particular interest the in region in which TESCO lies. 1.4 kb upstream of human TESCO, we discovered a locus containing a DNaseI hypersensitive site in embryonic testis and with strong active enhancer marks (Fig. 3a). Importantly, this region contains a putative SRY binding site and is highly conserved (Fig. 3a).

This 1259 bp fragment was significantly activated by co-transfection with SRY + SF1 or SOX9 + SF1, but did not respond to SOX9 alone (Fig. 1b). It showed significantly stronger transcriptional activation in vitro than human TESCO, which showed very little activity in this assay (Fig. 3b). Mutation of the SRY binding site significantly reduced activity when co-transfected with SRY + SF1 or SOX9 + SF1 (Fig. 3c), and addition of FOXL2 markedly repressed this activity (Fig. 3d). This 1259 bp human fragment shows 78% sequence conservation with mouse. CRISPR/Cas9 mediated deletion of the orthologous 1509 bp mouse sequence resulted in a significant reduction in *Sox9* expression levels at E11.5 and E14.5 (Fig. 3e). However, this did not alter the expression levels of *Amh, Wnt4 or Foxl2* (Supplementary Figure 7). Together, these data indicate that the 1259 bp fragment contains a third human enhancer important for SRY initiation of *SOX9* expression in the developing testis. We have designated this enhancer Alternate Long-Distance Initiator (eALDI) of *SOX9*.

### All three enhancers synergise to upregulate *SOX9* expression

**Fig. 4:**
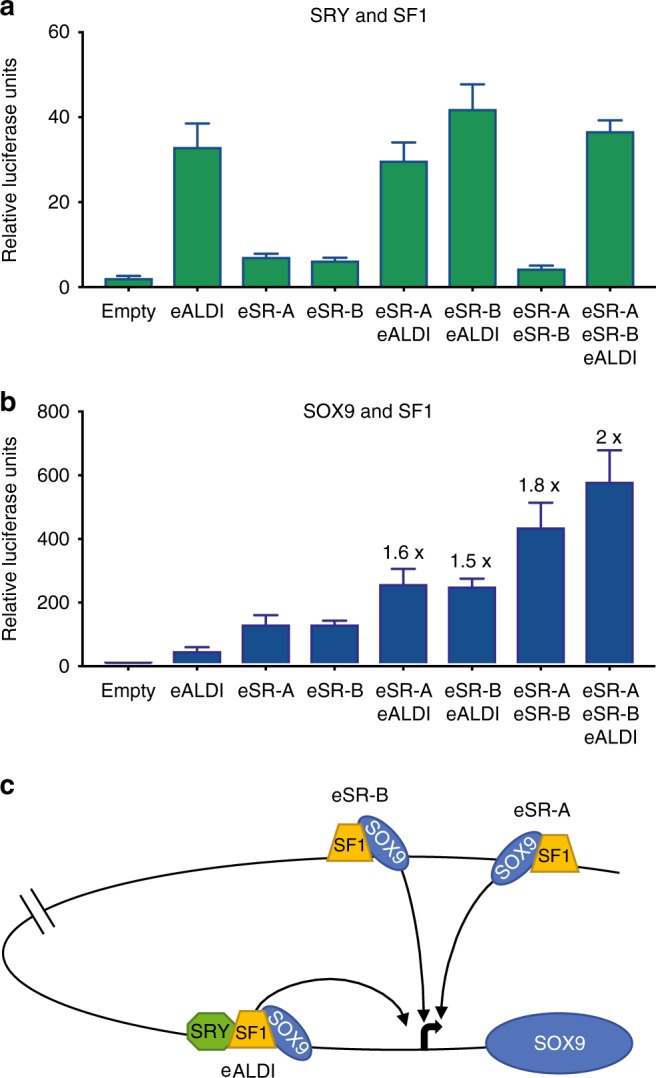
Testis-specific *SOX9* enhancer synergy and a proposed model for *SOX9* activation. Luciferase activity of three novel human *SOX9* enhancers individually and in tandem for **a** SRY + SF1 (green) and **b** SOX9 + SF1 (blue) in COS7 cells. *n* = 5, Error bars represent s.e.m. Fold activation was determined by average luciferase activity of the tandem construct, relative to the sum of the average luciferase activity of the individual enhancers. This value is shown above the error bars. **c** Proposed model of *SOX9* activation. In XY individuals SRY and SF1 are expressed early in the human XY foetal gonad, and initiate *SOX9* expression via the eALDI enhancer. *SOX9* expression is then further upregulated and maintained by SF1 and SOX9 with all three enhancers eSR-A, eSR-B and eALDI potentially via chromatin looping, allowing direct interaction between the enhancers and the *SOX9* promoter. Source data are provided as a Source Data file.

Previous reports of chondrocyte-specific *SOX9* enhancers have shown that interaction of multiple enhancers can lead to synergistic gene activation^29^. Therefore, we assessed whether this was also the case with our three gonadal enhancers. We cloned different combinations of enhancers (eSR-A, eSR-B and eALDI) in tandem and tested these in our luciferase reporter assay. SRY is the initiator of *SOX9* expression in the early gonad. In combination with SF1, SRY strongly activates the eALDI enhancer, but not eSR-A or eSR-B (Fig. 4a). eSR-A and eSR-B combined showed no response to SRY + SF1 (Fig. 4a) and when eALDI is present in tandem with eSR-A, eSR-B, or both, there was no synergistic increase in activity (beyond the accumulative total), suggesting that for initiation of *SOX9* expression by SRY, eALDI is the primary enhancer.

Following SRY initiation, SOX9 in combination with SF1 can regulate its own expression. Analysis of the tandem enhancers in the presence of SOX9 and SF1 shows that in pairs, eSR-A and eSR-B, eSR-A and eALDI, and eSR-B and eALDI all synergise (1.6, 1.5 and 1.8 fold respectively) (Fig. 4b). Together, all three enhancers showed the greatest synergy (2 fold) (Fig. 4b), suggesting that all three human enhancers have a combined role in the positive autoregulation of *SOX9* expression.

To confirm whether the three enhancers were active in a human chromatin context, we performed ChIP-PCR using H3K27ac, a marker for active enhancers^30^ on a human testis carcinoma cell line (NT2-D1) which, in the absence of human tissues, acts as a proxy for human embryonic testicular material. The ChIP revealed that eSR-A, eSR-B and eALDI are all present in active enhancer chromatin, with eSR-A and eSR-B showing strongest H3K27ac enrichment relative to the control (Supplementary Figure 8).

Together, our results suggest a model by which each enhancer has an essential yet distinct role in initiating and maintaining human *SOX9* expression. In this model, eALDI is the primary enhancer by which SRY and SF1 act to initiate *SOX9* expression; SOX9 alone or in combination with SF1 can then upregulate expression via all three enhancers, which may together act on the *SOX9* promoter through chromatin looping events (Fig. 4c).

## Discussion

In this study, we have identified human enhancers that contribute to *SOX9* expression, and in which genetic aberrations lead to sex reversal. The first two enhancers, eSR-A and eSR-B, were located using new patient CNV data. Both of these enhancers show activation by SOX9 alone or in combination with SF1, but little activation by SRY, the initiator of testis differentiation. eSR-A and eSR-B must logically be active in the absence of SRY in vivo, because their duplication results in *SOX9* overexpression in XX patients without SRY, ultimately causing XX sex reversal. Thus, in searching for an additional SRY-responsive enhancer, we identified eALDI. Indeed, our tandem enhancer analysis highlighted eALDI as the primary SRY-responsive enhancer. Therefore, we suggest that SRY and SF1, expressed early during human testis development, bind eALDI to initiate *SOX9* expression in the embryonic male gonad. Secondly, following this SRY-dependent initiation of *SOX9*, SOX9 and SF1 together bind sites within eSR-A, eSR-B and eALDI to amplify testicular *SOX9* expression. We saw the greatest enhancer synergy with activation by SOX9 and SF1 when all three enhancers were combined. Given the significant distance between these enhancers (63 kb between eSR-A and eSR-B and 555 kb between eSR-B and eALDI), it is probable that chromatin looping events between the enhancers and the *SOX9* promoter contributes to their synergistic regulation of *SOX9* expression (see model in Fig. 4c).

While it is difficult to know the exact sequence of events during activation of human *SOX9* in vivo, analysis of H3K27ac enhancer ChIP indicated that these three enhancer regions are active in an embryonic testis carcinoma cell line, NT2-D1. In addition, RoadMap data from 13-week-old human foetal testes or ovaries show that both eALDI and eSR-A have significant DNaseI peaks, suggesting open chromatin and functionality later in gonad development, while eSR-B does not. This may suggest sequential activity. Irrespective of the order of events, which is impossible to determine with certainty given the limitations of studying early human embryonic development, our combined data suggest that these three enhancers underlie the initiation, upregulation and auto-regulatory maintenance of *SOX9* in the testis during human sex determination. Recent analysis has demonstrated that gene regulation relies on multiple redundant enhancers more frequently than independent enhancer elements. Interestingly it is common to find three or more enhancers per gene^31,32^. Our data are consistent with these findings.

These human enhancers also show variable conservation in mouse. eSR-B drove reporter gene (LacZ) expression in mouse testis, and also strikingly in embryonic ovaries at different stages of development. It is possible that eSR-B drove gonad specific expression but require as yet unidentified repressor elements to ensure silencing in the ovary. A recent study has shown that loss of the mouse orthologue of eSR-A results in complete sex reversal (Enh13), demonstrating that this is a highly conserved essential testis enhancer^13^. We have shown that the mouse eSR-A enhancer is activated by mouse SRY and SF1 in luciferase assays, in contrast to the human enhancer eSR-A which does not respond to SRY, and is more likely involved in the upregulation and maintenance of SOX9. Thus, while both the human and mouse enhancers have a key role in testis-specific expression of *SOX9*, the way in which they are activated differs between the species. Unlike eSR-A, loss of function analysis of mouse eSR-B was uninformative and ChIP-seq for H3K27ac and Assay for Transposase-Accessible Chromatin using sequencing (ATAC-seq) analysis of E13.5 Sertoli cells in the mouse showed no potential enhancer elements in the mouse equivalent eSR-B^13,33^. As mouse eSR-B shows some enhancer activity in luciferase assays, mouse eSR-B may have a redundant or shadow enhancer role in mice at a time point yet to be examined by ChIP or ATAC-seq.

In mice, Testis Specific Enhancer of Sox9 (TES) with its 1.4 kb core, TESCO, lies 13 kb upstream of the *Sox9* gene, and can enhance *Sox9* expression in response to SF1, SRY and SOX9 in mice. By contrast, we have shown in vitro that sequences homologous to TES/TESCO do not appear to play a role in human *SOX9* regulation. Instead, the functional characteristics of human eALDI show a striking resemblance to those of TESCO in mice. Both are activated most strongly by SOX9 + SF1, and to a lesser extent, SRY + SF1 or SF1 alone^11^. Both eALDI and TESCO are important for *Sox9* expression levels but are not critical for male sex determination in mice, as revealed by CRISPR/Cas9 targeting. We found a 60% reduction of *Sox9* at 11.5 dpc in the eALDI null mice which is very similar to that observed in the TESCO/TES mutant mice, which express *Sox9* at 62 and 44% of wild-type *Sox9* levels, respectively, with no sex reversal^12^. Threshold levels for *SOX9* vary between human and mice as human patients heterozygous for *SOX9* mutations often display 46,XY DSD sex reversal while mice lacking one copy of *Sox9* do not^12^. It is possible that the functional similarities between eALDI and TESCO may mean that eALDI is the TESCO equivalent in humans/primates. To date we have not identified CNVs in the eALDI region in DSD patients, yet given the close proximity of eALDI to TESCO and the *SOX9* gene, any CNV would likely affect both enhancers and/or the gene itself^34^. However, we will continue to screen DSD patients for aberrations in this region.

In summary, this is the first report of human testis-specific enhancers that, if duplicated or deleted lead to 46,XX or 46,XY DSD (sex reversal), respectively, with no effect on other developmental processes that require *SOX9* (i.e absence of skeletal abnormalities or other features of campomelic dysplasia). For both eSR-A and eSR-B, loss of one copy in 46,XY DSD patients appears sufficient to prevent upregulation or maintenance of *SOX9* expression to the levels required to ensure proper testis development. By contrast, a single additional copy of either of these enhancers promotes the expression of *SOX9* to a level that is sufficient to override the ovarian programme causing 46,XX testicular or ovotesticular DSD. We envisage that this occurs through the activation of the duplicated enhancer(s) by SF1 and basal levels of SOX9 present in the XX foetal gonad^35^ to ultimately upregulate *SOX9* and drive Sertoli cell differentiation while repressing the ovarian pathway. The difference we observe in clinical phenotypes could be due to individual differences in the basal level of SOX9, or to additional genetic modifiers.

In humans, the molecular network underlying gonad sex determination is not well understood. This fundamental lack of understanding impacts on our ability to diagnose patients with DSD. Currently, only 38.7% of patients receive a genetic diagnosis based on the limited number of genes with pathogenic variants^36^. This implies that alteration in novel genes or regulatory regions may explain the remaining undiagnosed DSD patients. eALDI, eSR-A and eSR-B enhancers are present within a “gene desert” that is often excluded from genetic screens or arrays. This study shows that these newly defined regions should be included in any diagnostic assays for unexplained cases of DSD, especially SRY-negative 46,XX testicular or ovotesticular DSD or 46,XY DSD. Our data provide a new and experimentally supported model for *SOX9* regulation in humans and may provide the long sought-after missing link between SRY, SF1 and *SOX9* initiation, upregulation and maintenance, all of which are required for testis and ultimately human male development.

## Methods

### Patient recruitment and ethics

All patients gave informed consent under the Melbourne Royal Children’s Hospital ethics application HREC22073 as previously described^36^. All genomic co-ordinates refer to human genome hg19.

### CGH-array mapping of CNVs

A custom 60K oligo CGH microarray was designed on the SureDesign software and manufactured by Agilent Technologies (Santa Clara, CA), using GRCh37/hg19. Probes were selected to detect CNVs at exon level for a defined number of genes known to cause DSD (van den Bergen, unpublished). Some *SOX9* regulatory regions were also targeted (CHR17:69475000-69560000 (*RevSex*) and CHR17:70102435-70105514 (*TESCO*)), to detect CNVs at a functional resolution of 500 bp. An approximate whole genome backbone resolution of 120 kb was maintained. Patient and reference DNA were processed as specified by manufacturer protocols. Computational analysis was performed using CytoGenomics (Agilent), CGH algorithms ADM2 used at a threshold of 6.0.

### Reporter constructs and luciferase assays

Analysis of the minimal overlap of multiple patient CNV upstream of SOX9 identified two genomic regions of interest. The minimal region of interest location within XYSR 5.2 kb was tiled targeting DNaseI hypersensitive regions predicted to be present in human foetal testis. The minimal region of interest for RevSex is 24 kb an unbiased tiling array was used to span the entire region of interest, generating 16 overlapping ~2 kb fragments. The eALDI enhancer was located using targeted bioinformatic screening of the upstream region of SOX9 using multiple datasets, ENCODE^27^, RoadMap^23^, transcription factor binding site and vertebrate conservation^37^, focusing on regions that showed enhancer function in all three datasets.

All genomic fragments (Supplementary Table 1) were amplified from human genomic DNA using Phusion polymerase (NEB) and cloned into the luciferase reporter vector pGL4.10 (Promega) containing the human beta-globin minimal promoter^38^ between bgIII and HindIII sites in the multiple cloning site. Transcription factor consensus binding sites (SF1, SOX9 etc) were identified using JASPER motif analysis software^39^ and mutated using the Quikchange site directed mutagenesis kit II XL (Agilent) (see Supplementary Table 1 for primers). All luciferase assays were performed in COS7. Briefly, cells were seeded at 80–90% confluency in 96 well plate for 2–4 h. Each well was transfected with a combination of enhancer reporter plasmid pgl4-beta-globin (75 ng) and transcription factors in pcDNA3.1 (100 ng) with 15 ng of Renilla luciferase pLR-TK (Promega) as a control using lipofecamine 2000 for 24 h. The cells were lysed using 1.25x passive lysis buffer (Promega) and luciferase was determined using Dual-Luciferase® Reporter Assay System (Promega) as previously described^40^. All enhancers were further tested in HEK293T, TM3 and TM4 using the same method as COS7 (Supplementary Figure 2).

### Luciferase assay data analysis

For all luciferase assays we normalised the Firefly luciferase values to the Renilla luciferase values (control for transfection efficiency) and then to the average of the empty transcription factor vector (pcDNA3.1) control. All experiments were carried out in duplicate. We calculated standard error of the mean (s.e.m.) and two-tailed Students *t*-test from at least three biological replicates (independent transfections) using Graphpad Prism V7.

### Mouse studies

All animal work was conducted according to protocols approved by the University of Queensland Animal Ethics Committee. Embryos were collected with noon of the day a mating plug was observed designated 0.5 days post coitum (dpc). Chromosomal sex was determined by PCR genotyping as described^41^.

### Enhancer-*lacZ* transgenics for eALDI and eSR-B

Transgenic constructs were prepared by cloning the putative enhancer (eALDI and eSR-B) in front of a human β-globin proximal promoter and the *lacZ* reporter. Transient transgenic mice were prepared by standard^42^ or piggybac-assisted^43^ means and gonads were collected and analysed between E11.5-E14.5. In the case of eSR-B and eALDI stable transgenic lines were also generated (a total of 11 and 3, respectively) and gonads were isolated from founder offspring embryos at various time points and analysed. One eSR-B-*lacZ* line robustly expressed the transgene and was analysed further; none of the three eALDI lines showed expression.

### β-galactosidase staining

Transient transgenic gonads with associated mesonephroi from control embryos, or from the eSR-B-lacZ line at various time points, were stained for β-galactosidase activity using standard methods and incubating overnight at 37 °C, as previously described^44^.

### Sequence conservation of the three enhancers

Sequence conservation analysis (www.ncbi.nlm.nih.gov/BLAST/) of the 1514 bp human eSR-A sequence to the mouse genome identified that eSR-A (hg19, chr17:69480137-69481650) aligned to mm9 chr11:112077985-112079421 with 80% sequence conservation.

Sequence conservation of the 1259 bp human eALDI sequence, located 16 kb 5′ to *SOX9* (*Homo sapiens* chromosome 17: 70,100,240–70,101,498: ref NC00017.11), identified a 1176 bp sequence of 76% similarity located 14.4 kb 5′ to mouse *Sox9* (*Mus musculus* strain C57BL/6J, chromosome 11: 112,766,990–112,768,165: ref NC000077.6).

Sequence conservation of the 402 bp human eSR-B sequence, located 572 kb 5′ to SOX9 (*H. sapiens* chromosome 17: 71,548,507–71,548,908: ref NC00017.11), identified a 392 bp sequence of 75% similarity located 511 kb 5′ to mouse Sox9 (M. musculus strain C57BL/6J chromosome 11: 112,270,933–112,271,324: ref NC000077.6).

### Mutation of mouse eALDI and eSR-B

Using the homology analysis described above two 18-mer single guide RNAs (sgRNAs) were designed (GT-Scan, http://gt-scan.braembl.org.au,) to flank and delete the 1176 bp mouse sequence by CRISPR/Cas9 editing^45^. A perfect excision between the two targeted Cas9 cleavage sites would remove 1368 bp of sequence encompassing the 1176 bp mouse ‘eALDI’ sequence.

Using the homology analysis described above two 20-mer single guide RNAs (sgRNAs) were designed (www.crispr.mit.edu/) to flank and delete the 392 bp mouse eSR-B sequence by CRISPR/Cas9 editing. A perfect excision between the two sgRNA target cleavage sites would remove 541 bp of sequence encompassing the 392 bp mouse eSR-B element.

sgRNAs and Cas9 mRNA were prepared as described elsewhere^46^. Briefly, for each sgRNA the complementary pair of oligos (Extended Data Table 2) was annealed and cloned into pX330 (Addgene #42230). A T7-sgRNA PCR product was amplified with a T7 promoter sequence introduced on the forward primer in conjunction with a universal reverse primer sgRNA-uni.R (Supplementary Table 2) This product was used as the template for in vitro transcription (IVT) using the MEGAshortscript T7 IVT kit (Life Technologies). The Cas9 coding region was released from pX330 and sub-cloned into pBluescript II (pBS-Cas9). *Xho*I linearised pBS-Cas9 was used as the template for IVT using the mMESSAGE mMACHINE T7 ULTRA kit (Life Technologies). Both sgRNAs and Cas9 mRNA were purified using the MEGAclear kit (Life Technologies) and eluted in RNase-free water. 30 ng/μl of Cas9 mRNA and 15 ng/μl of each sgRNA diluted in RNase-free water were injected into one pronucleus of C57BL/6 × CBA F1 hybrid one-cell embryos. Injected embryos were cultured overnight to the two-cell stage, then surgically transferred into oviducts of day-of-plug pseudopregnant CD1 mice.

Transgenic founders with a full deletion of the eSR-B and eALDI sequence were identified amongst live born mice by PCR genotyping (all genotyping primers are listed in Supplementary Table 2). All genotyping was performed using genomic DNA extracted from tail tissue of embryos or ear notch tissue of juvenile animals. Male founders were bred to C57BL/6 females to verify that deleted alleles were germline transmitted to progeny. eSR-B and eALDI deletions were verified by cloning and sequencing of amplified products from heterozygous offspring of founders. Stable lines were established from two male founders (eSR-B), one bearing a 502 bp deletion and the other bearing a 566 bp deletion, both of which encompassed the eSR-B element. While one male founded eALDI bearing a 1509 bp deletion. For all embryos and pups, chromosomal sex was determined by PCR genotyping with primers Sex-F and Sex-R (Supplementary Table 2)^41^.

### qRT-PCR to assess CRISPR knockouts

Gonadal tissue was isolated and subjected to RNA synthesis (Qiagen RNeasy micro kit including DNase treatment) and cDNA synthesis (Applied Biosystems (ABI), High Capacity cDNA Archive kit). Relative cDNA levels were determined by the 2^−ΔCT^ method with reactions including Taqman PCR master mix (ABI) and Taqman gene expression sets and carried out on an ABI ViiA7 machine. The control gene used for normalisation was *Tbp* (encoding the ubiquitously expressed TATA box binding protein, Mm00446973_m1) Primers are listed in Supplementary Table 2. Statistical significance was determined using Prism GraphPad software (*t*-test).

### Haematoxylin and eosin (H&E) and immunofluorescence (IF)

Whole embryos were recovered and IF analyses were carried out on fixed, paraffin-embedded 7 mm sections using standard methods as detailed previously^47^. H&E histological staining was performed on 7μm-thick sagittal sections (E14.5 and 6 weeks) according to standard protocols. IF was conducted using high pH antigen retrieval and blocking in 10% heat inactivated horse serum in PBTX. Primary antibodies used were: goat anti-AMH (1:500, Santa Cruz Biotechnology), rabbit anti-FOXL2 (1:650, generated as described previously^48^), mouse anti-MVH (1/600; code ab27591, Abcam), goat anti-FOXL2 (1/200; code ab5096, Abcam), rabbit anti-SOX9 (1/200; code AB5535, Millipore), chicken anti-b-galactosidase (1/200; code ab9361, Abcam). Secondary antibodies (all from Molecular Probes, Invitrogen) were goat anti-mouse IgG Alexa Fluor 594, goat anti-rabbit IgG Alexa Fluor 488, and goat anti-chicken IgY Alexa Fluor 488, donkey anti-rabbit Alexa Fluor 647, donkey anti-goat Alexa Fluor 488. Slides were imaged on an Olympus BX-51 fluorescence microscope.

### Chromatin immunoprecipitation

The human Sertoli-like cell line NT2/D1 was cultured in DMEM/F12 medium (Invitrogen, Carlsbad, CA, USA) with 10% foetal bovine serum and 1% penicillin-streptomycin at 37 °C with 5% CO_2_. Cells (5 × 10^7^) were detached with Versene Solution (Life Technologies), resuspended in 1X PBS, pelleted by centrifugation, and resuspended in 20 ml Fixing Buffer (50 mM HEPES-KOH pH 7.5, 100 mM NaCl, 1 mM EDTA pH 8.0, 0.5 mM EGTA, pH 8.0). All solutions were supplemented with 1× protease inhibitor cocktail (Roche, Basel, Switzerland). Cells were fixed for 10 mins at room temperature in 1% formaldehyde and the reaction quenched with 125 mM glycine for 5 min at room temperature. Cells were pelleted by centrifugation at 4 °C and resuspended in 5 ml cold 1× PBS. Cells were again pelleted and resuspended in 10 ml lysis buffer LB1 (50 mM HEPES-KOH, pH 7.5, 140 mM NaCl, 1 mM EDTA, 10% glycerol, 0.5% NP-40, 0.25% Triton X-100). After lysis, cells were pelleted and resuspended in 10 ml LB2 (10 mM Tris–HCl pH 8.0, 200 mM NaCl, 1 mM EDTA pH 8.0, 0.5 mM EGTA pH 8.0). Nuclei were pelleted by centrifugation and resuspended in 1 ml LB3 (10 mM Tris–HCl pH 8.0, 200 mM NaCl, 1 mM EDTA, pH 8.0, 0.5 mM EGTA pH 8.0, 0.1% Na-deoxycholate, 0.5% *N*-lauroylsarcosine). Centrifugation and resuspension of nuclei in 1 ml LB3 was then repeated. Nuclei were finally pelleted and resuspended in 3 ml LB3. The chromatin was sheared by a Covaris S220 ultrasonicator (Covaris, Inc., Woburn, MA, USA) at 4 °C for 10 min (duty cycle: 10%, intensity: 8, 200 cycles per burst, power mode: frequency sweeping). Sheared samples were centrifuged at 14,000 rpm for 1 min at 4 °C and cell debris was discarded.

Immunoprecipitation of sheared chromatin was then performed with rabbit H3K27ac (Abcam ab4729) and goat anti-rabbit IgG (Sigma-Aldrich R2004). 50 µl Dynabeads (Invitrogen) was incubated with BSA (220 µg), sonicated herring sperm DNA (40 µg) and either 10 µg H3K27ac antibody or 10 µg IgG for 3.5 h at 4 °C on a rocker. The antibody-Dynabeads complex was incubated with 500 µl sheared chromatin for 18 h at 4 °C, washed with ChIP RIPA buffer (10 mM Tris-Cl pH 7.6, 1 mM EDTA, 0.1% SDS, 0.1% sodium deoxycholate, 1% Triton X-100) and then washed with 1× PBS. Protein–DNA complexes were eluted with 500 µl elution buffer (0.1 M NaHCO_3_, 1% SDS) by incubation for 30 min at room temperature. To reverse cross-link protein–DNA complexes, samples were incubated with 450 mM NaCl for 18 h at 65 °C. Protein and RNA were removed by incubation with 100 mM Tris pH 6.5, 25 mM EDTA and 50 µg Proteinase K for 1 h at 45 °C and by RNase treatment (Qiagen Inc., Valencia, CA, USA), respectively. DNA was purified using the QIAquick PCR Purification Kit (Qiagen). DNA quality and quantity were assessed using gel electrophoresis and a Nanodrop spectrophotometer (Thermo Fisher Scientific, Waltham, MA, USA).

### ChIP-PCR analysis

The purified immunoprecipitated DNA was subjected to PCR analysis to identify the degree of histone H3K27ac-binding within eSR-A, eSR-B and eALDI. DNA extracted from chromatin fractions prior to immunoprecipitation (i.e. input DNA) was included in the ChIP analysis. Primers were designed using Primer-BLAST to amplify ~150 bp products^49^ (Supplementary Table 3). Exon 1 of the *GAPDH* gene was used as a positive control (primers from Abcam plc, Cambridge, UK), as it has high levels of histone modifications associated with active gene transcription and low levels of histone modifications associated with gene silencing. A negative control for the PCR reactions with no template added was also included. PCR products were electrophoresed on a 2% agarose gel and imaged using a Gel Doc XR+ System (Bio-Rad Laboratories, Inc., Hercules, CA, USA).

## Supplementary information

Supplementary Information

Source Data

## Data Availability

CGH-array patient data have been submitted to NCIB ClinVar. Patient 1 accession SCV000845738, Patient 2 accession SCV000845739. All unique materials and data are readily available from the authors. The source data underlying Figs. 1d–f, 2e–g, 3b–f, 4a, b and Supplementary Figures 2, 3, 5, 6, 7 and 8 are provided as a Source Data file.
