# Author Correction: Human sex reversal is caused by duplication or deletion of core enhancers upstream of *SOX9*

**DOI:** 10.1038/s41467-019-11310-w

**Published:** 2019-07-23

**Authors:** Brittany Croft, Thomas Ohnesorg, Jacqueline Hewitt, Josephine Bowles, Alexander Quinn, Jacqueline Tan, Vincent Corbin, Emanuele Pelosi, Jocelyn van den Bergen, Rajini Sreenivasan, Ingrid Knarston, Gorjana Robevska, Dung Chi Vu, John Hutson, Vincent Harley, Katie Ayers, Peter Koopman, Andrew Sinclair

**Affiliations:** 1 Murdoch Children’s Research Institute, Melbourne, 3052 VIC Australia; 2 Department of Molecular & Translational Science, Monash University, Clayton, 3800 VIC Australia; 3 Department of Paediatrics, The University of Melbourne, Melbourne, 3010 VIC Australia; 4 School of Bioscience, University of Melbourne, Melbourne, 3010 VIC Australia; 5 Department of Paediatric Urology, Monash Children’s Hospital, Clayton, 3168 VIC Australia; 6 School of Biomedical Sciences, University of Queensland, Brisbane, 4072 QLD Australia; 7 Institute for Molecular Bioscience, The University of Queensland, Brisbane, 4072 QLD Australia; 8 Bioinformatics Division, Walter & Eliza Hall Institute of Medical Research, Melbourne, 3052 VIC Australia; 9 Hudson Institute for Medical Research, Clayton, 3168 VIC Australia; 10 Department of Medical Genetics, Metabolism & Endocrinology, National Children’s Hospital, Hanoi, Vietnam; 11 Department of Urology, Royal Children’s Hospital, Melbourne, 3052 VIC Australia

**Keywords:** Gene expression, Gene regulation

Correction to: *Nature Communications* 10.1038/s41467-018-07784-9, published online 14 December 2018.

The original version of this Article contained an error in the spelling of the author Jacqueline Hewitt, which was incorrectly given as Jacky Hewitt. This has now been corrected in both the PDF and HTML versions of the Article.

