# Supplementary material for: Human sex reversal is caused by duplication or deletion of core enhancers upstream of SOX9
Source: Nat Commun. 2018 Dec 14;9:5319. doi: 10.1038/s41467-018-07784-9 (PMC6293998; doi:10.1038/s41467-018-07784-9)
Supplement: Supplementary file 1 — Supplementary Information [file 41467_2018_7784_MOESM1_ESM.pdf]

Human sex reversal is caused by duplication or deletion of core enhancers upstream of *SOX9*

Croft et al.

Supplementary Information

Supplementary Figures 1-8

Supplementary Tables 1-3

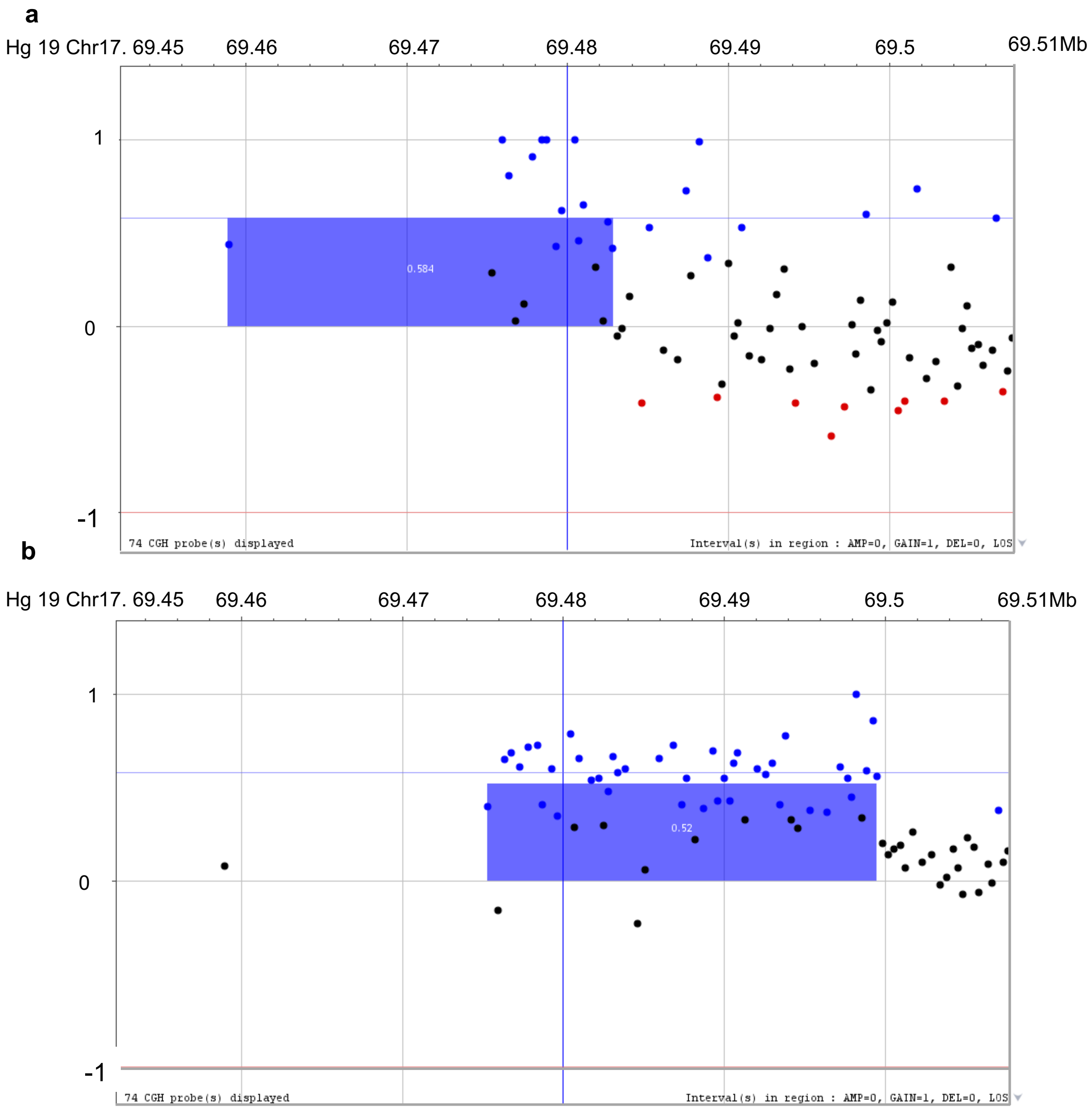

Supplementary Figure 1. CGH array identifies duplications upstream of *SOX9* in two 46,XX DSD patients. Results of the custom CGH (comparative genomic hybridization) array covering the SOX9 regulatory region for 46,XX DSD patients. Log2 scatter plots show individual data points, and the highlighted blue boxes represent the minimal gain regions. (a) Patient 1; ISCN: arr17q24.3(69,393,372x2,69,458,883-69,482,850x3,69,483,073x2) min/max EndFragment. Min: 23.9kb duplication, max: 89.7kb. Aberration calculated p-value 1.55x10-18. 3’ end is in the custom region breakpoint can be mapped within 223bp. 5’ breakpoint outside the custom more difficult to define (reflected in the max size). (b) Patient 2. ISCN: arr17q24.3(69,458,943x2,69,475,275-69,499,520x3,69,499,834x2) min/max. Min: 24.2kb duplication, max: 40.9kb. 3’ end within the custom region breakpoint can be mapped within 314bp. 5’ breakpoint outside of the custom region more difficult to define. Aberration calculated p-value 2.8x10-62.

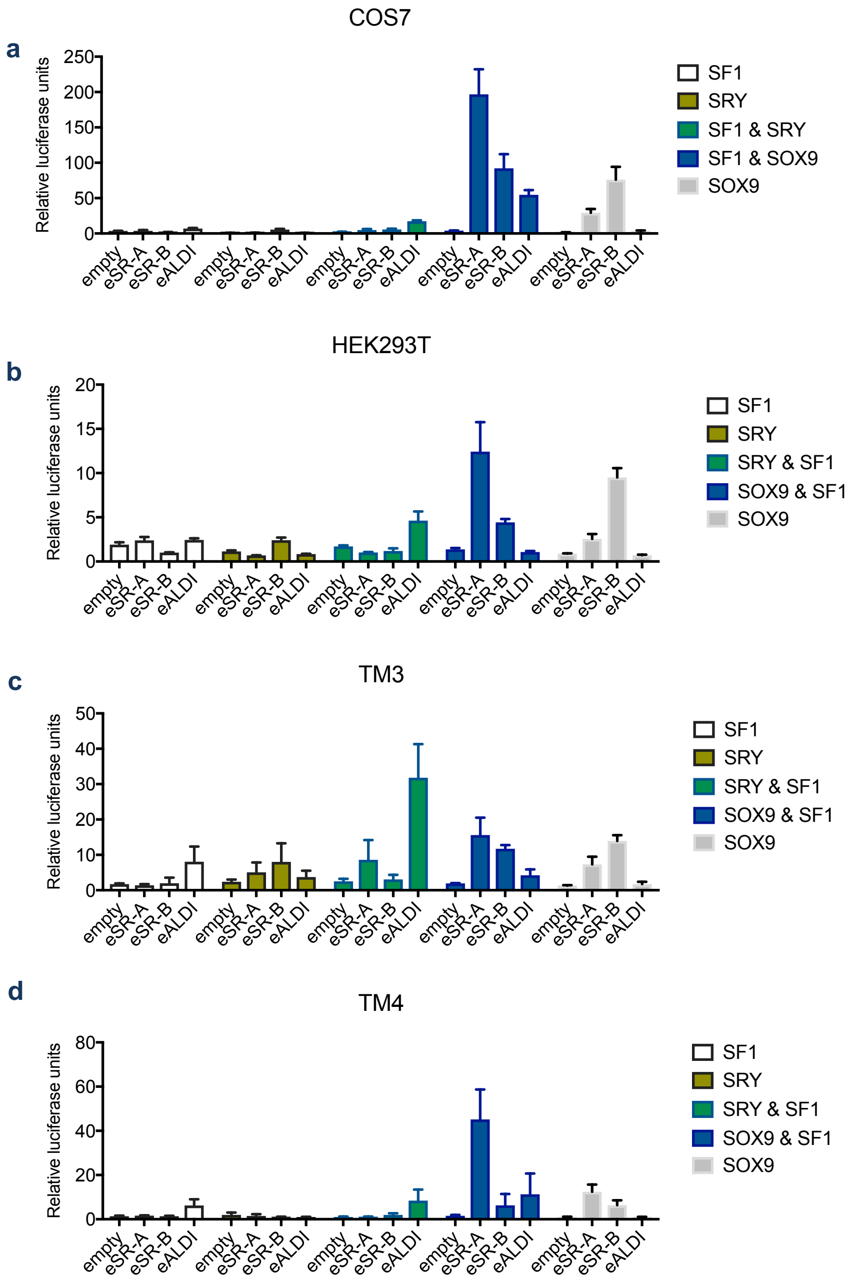

Supplementary Figure 2. Comparison of SOX9 enhancers in four cells lines

(a) *In vitro* luciferase activity of three SOX9 enhancers in the green monkey kidney cell line COS7 in the presence of transcription factors SF1, SRY and SOX9. (b) *In vitro* luciferase activity of three SOX9 enhancers in the human embryonic kidney cell line HEK293T in the presence of transcription factors SF1, SRY and SOX9. (c) *In vitro* luciferase activity of three SOX9 enhancers in the mouse Leydig cell line TM3 in the presence of transcription factors SF1, SRY and SOX9. (d) *In vitro* luciferase activity of three SOX9 enhancers in the mouse Sertoli cell line TM4 in the presence of transcription factors SF1, SRY and SOX9. N=3-4, error bars represent SEM. Source data are provided as a Source Data file.

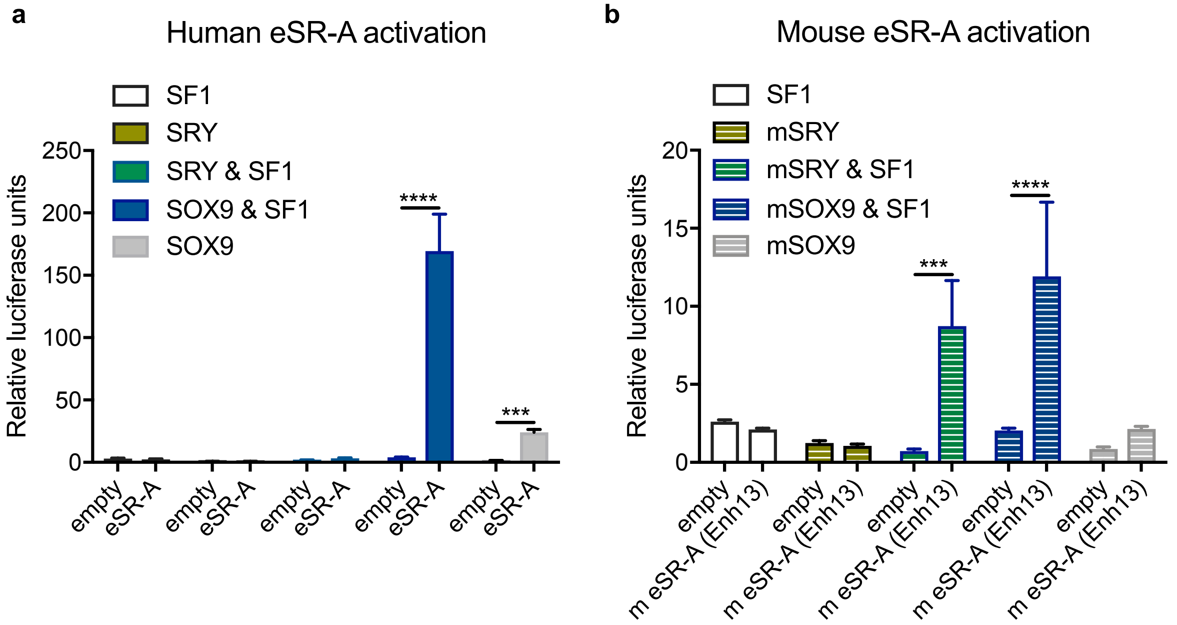

Supplementary Figure 3. Mouse and human SR-A enhancers are activated by different transcription factors. (a) *In vitro* luciferase activity of human eSR-A in COS-7 cells in response to human transcription factors SF1, SRY and SOX9. Significant activation is seen only with SOX9 alone or with SF1. (b) Activity of the mouse eSR-A ortholog (Enh13) with SF1, mouse SRY and mouse SOX9. Unlike human eSR-A, mouse eSR-A shows activation in response to mouse SRY with SF1. N=3-4, error bars represent SEM, p values determined by student t- test compared to the empty control, ***$p<0.001$ and ****$p<0.0001$. Source data are provided as a Source Data file.

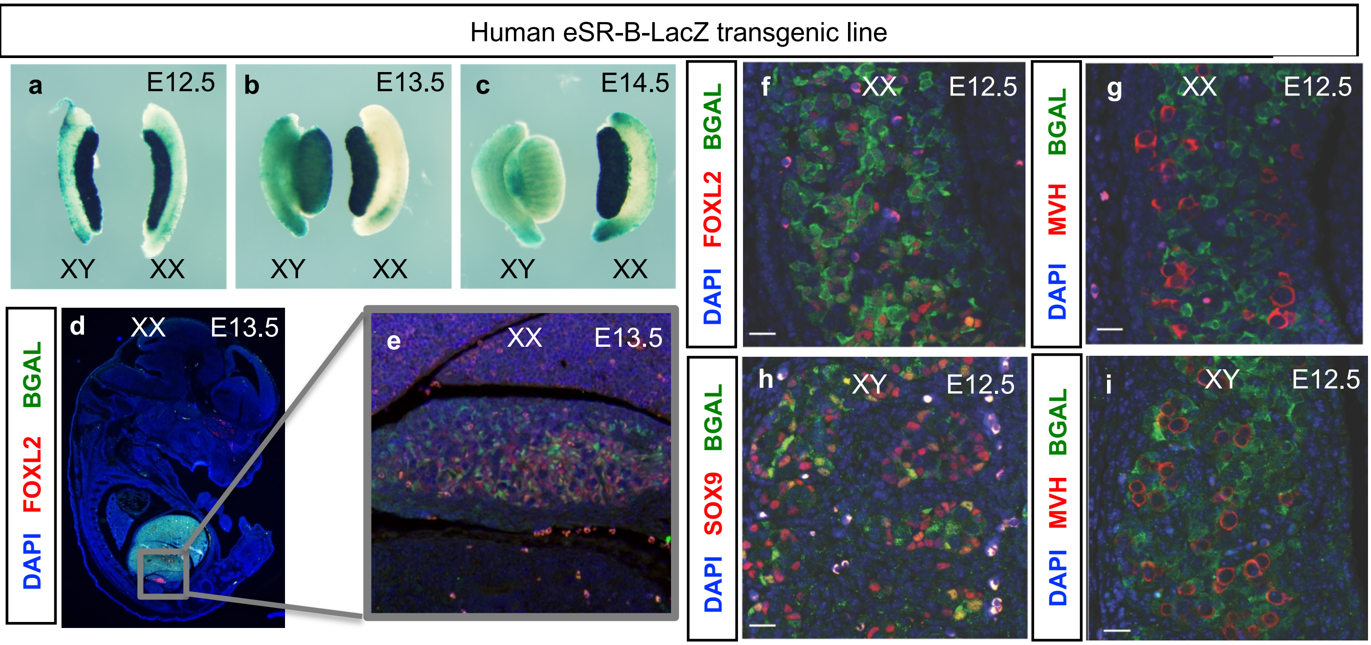

Supplementary Figure 4. Human eSR-B enhancer drives expression in the mouse embryonic gonads. Human eSR-B-BetaGlobin-LacZ expression. 11 transgenic lines were generated. A single representative stable transgenic line is presented here. In a time course, LacZ expression is strong at E12.5 in both ovaries and testis (a), but diminishes in the testis starting at E13.5 (b) and up to E14.5 (c). Expression is maintained in the ovary. A transverse section shows specificity to the gonad at embryonic day 13 (d and e). Expression in the ovary is seen in the somatic cells, overlapping with Foxl2 (f), and not the germ cells, stained with Mvh (g). In the testis, human eSR-B drives expression in the cords, overlapping with SOX9 (h), and not in the germ cells (i).

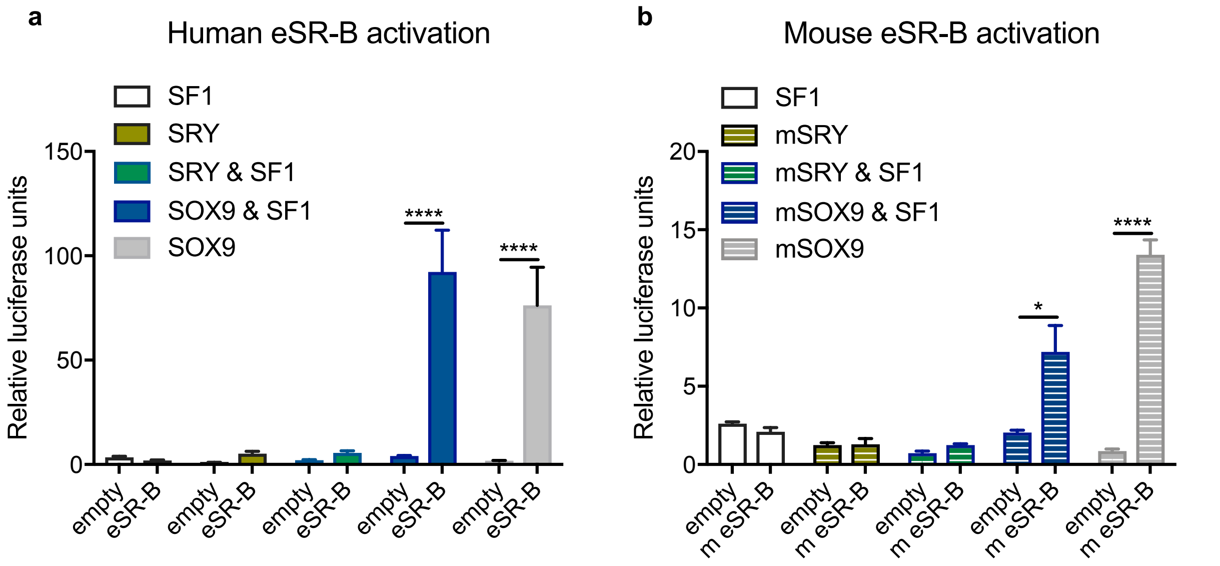

Supplementary Figure 5. Mouse and human SR-B enhancers are activated by the same transcription factors. (a) *In vitro* luciferase activity of human eSR-B in COS-7 cells in response to human transcription factors SF1, SRY and SOX9. Significant activation is seen only with SOX9 alone or with SF1. (b) Activity of the mouse eSR-B ortholog with SF1, mouse SRY and mouse SOX9. Mouse eSR-B shows significant activation is seen only with mouse SOX9 alone or with SF1. N=3-4, error bars represent SEM, p values determined by student t- test compared to the empty control, *$p<0.5$ and ****$p<0.0001$. Source data are provided as a Source Data file.

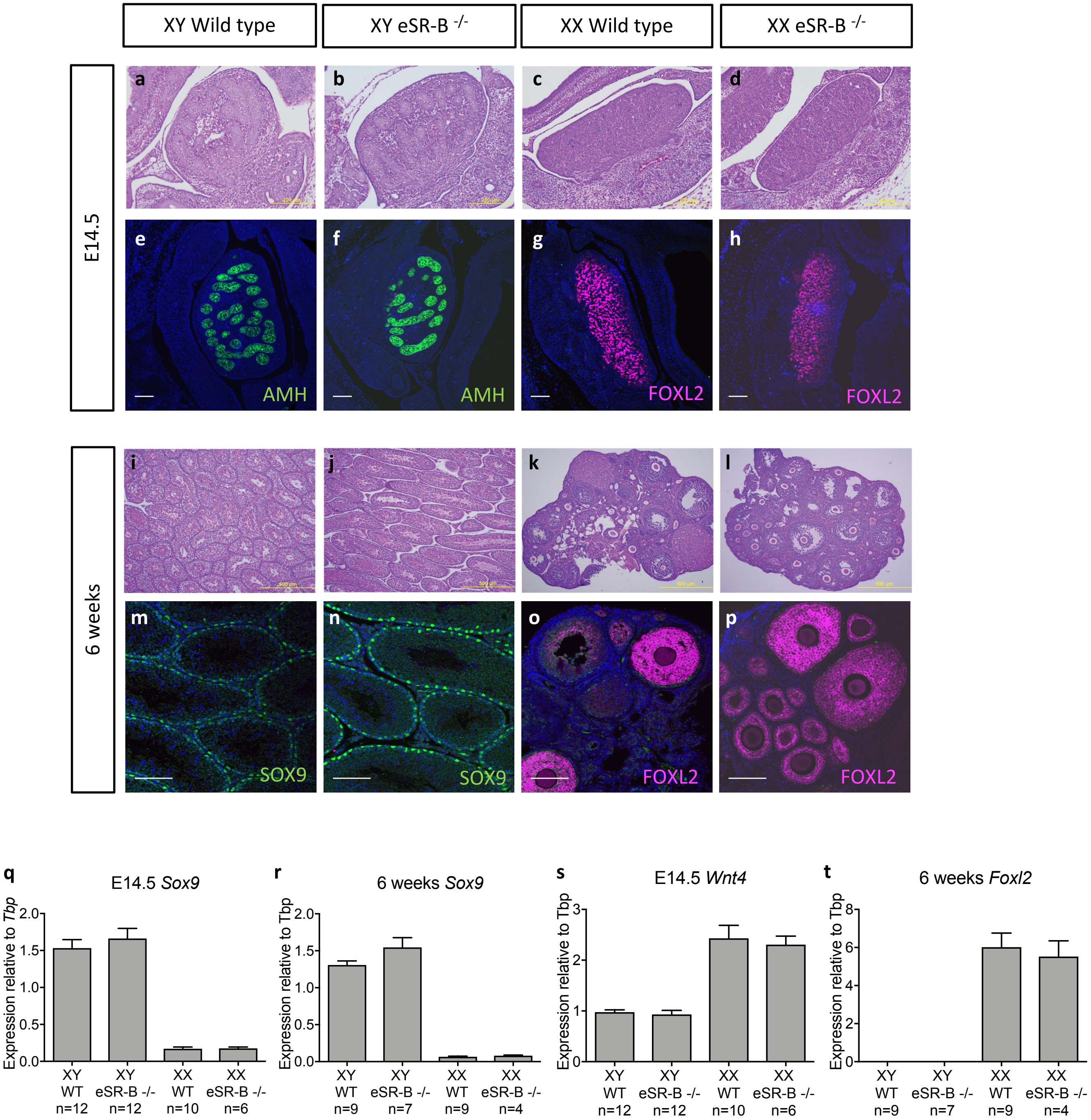

Supplementary Figure 6: Deletion of eSR-B in XY and XX mice does not affect gonad morphology or expression of sex determining genes. (a-d) Haematoxylin and eosin histological stains of sagittal sections for E14.5 embryos. Scale bar represents 500µm, n=1. (e, f) Immunofluorescence stains with the testis marker AMH (green) or (g, h) the ovarian marker FOXL2 (magenta), and DAPI (blue, e-h). Scale bar represents 100µm, n=2. (i-l) Haematoxylin and eosin histological stains of sagittal sections for 6 week gonads. Scale bar represents 500µm, n=1. (m, n) Immunofluorescence staining with the testis marker SOX9 (green), (o, p) the ovarian marker FOXL2 (magenta), and DAPI (blue, m-p). Scale bar represents 100µm, n=1. (q-t) qRT-PCR analysis of E14.5 gonads and 6 week postnatal gonads indicates no significant effect of eSR-B deletion on *Sox9* expression levels (q, r), or on marker genes for ovarian development or maintenance *Wnt4* (s) and *Foxl2* (t). Error bars represent SEM . Relative expression is presented as mean 2-ΔCt values for single gonads from multiple individuals (samples sizes as indicated on charts). Source data are provided as a Source Data file.

Supplementary Fig. 6

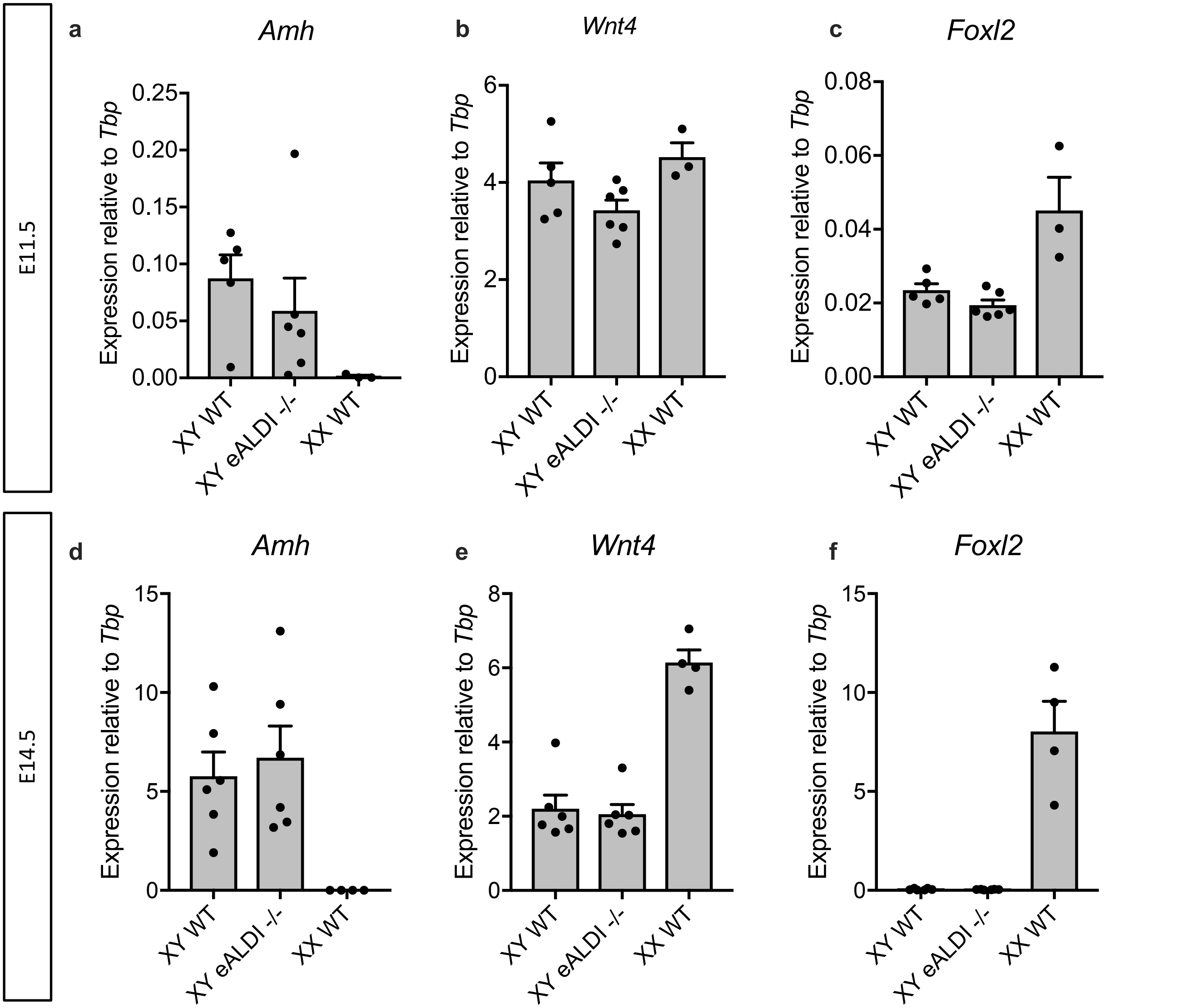

Supplementary Figure 7: Deletion of ALDI in the mouse does not affect the expression of sex determining genes with the marked exception of *Sox9* (See Fig. 3e,f). qRT-PCR analysis of E11.5 (a-c) and E14.5 (d-f) mouse gonads. Compared to XY wild type mice (WT), ALDI deletion causes no significant change in expression of *Amh* at E11.5 (a) or E14.5 (d). Markers for ovarian development (Wnt4, b, e) or maintenance (*Foxl2, c, f*) are not increased in ALDI mutant gonads, compared to XY wildtype. scale bars represents +/- SEM. Relative expression is presented as mean 2-ΔCt values for single gonads from multiple individuals (samples sizes as indicated on charts, one dot per biological repeat). Source data are provided as a Source Data file.

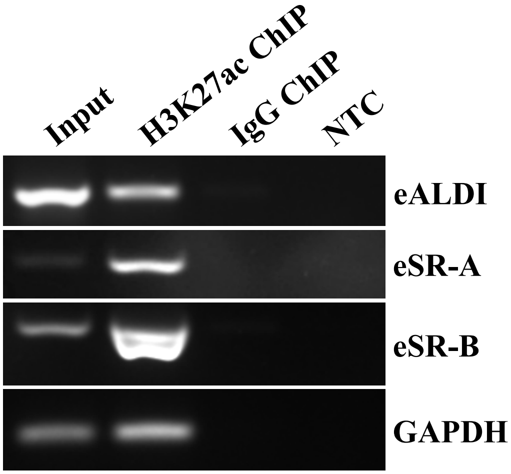

Supplementary Figure 8: ChIP analysis to determine the chromatin state of the eALDI, eSR-A and eSR-B enhancers in NT2/D1 human Sertoli-like cells. The eALDI, eSR-A and eSR-B enhancer regions were analysed by ChIP using an H3K27ac antibody that marks active enhancer regions. Input chromatin, H3K27ac- and control IgG-immunoprecipitated DNA and a no template control (NTC) are shown. Primers were designed to PCR amplify ~150 bp fragments from the three enhancers. The GAPDH gene was included as a positive control. Source data are provided as a Source Data file.

Supplemental Table 1 ***Genomic location and oligos used for cloning luciferase fragments***

Name	hg19 Chr:17	Forward primers	Reverse primers	Notes
XYSR				
a1	69,478,600-69,483,197	TTGATGGTAGACCTCGTGGTACC	ACAGCTAGCGTTATTTGTTCTTTACATCTGG	
a2	69,478,600-69,480,242	TTGATGGTAGACCTCGTGGTACC	BGLII DIGEST	
a3	69,480,137-69,483,197	AAAGGTACCAATCTCATTGACCACTCAGAATTG	ACAGCTAGCGTTATTTGTTCTTTACATCTGG	
eSR-A	69,480,137-69,481,650	AAAGGTACCAATCTCATTGACCACTCAGAATTG	TTTGCTAGCCTACTGCATCTTACCTACTGTG	
a5	69,480,745-69,481,358	AAAGGTACCTTCATAGAAATAATCTGCTGTGTTGC	TTTGCTAGCTCAACTTGGAGTCTTCAGTTTGC	
eSR-A ΔSOX9		TTTCAGAGTTTTTATCACAGTTCTTATCCCGTGGTCTGTTTTCACAAGCCAATGTG	CACATTGGCTTGTGAAAACAGACCACAGGGATAAGAACTGTGATAAAAACTCTGAAA	ACA>GGG
eSR-A ΔSF1-a		ATGAAACTATTTTCACTTTTCTACGAAAGGGGCAATAGGGG	CCCCTATTGCCCCTTTCGTAGAAAAGTGAAAATAGTTTCAT	AAGG>DEL
eSR-A ΔSF1-b		CACAAGCAGCTGGAAGGGAGTGCTTTTATGTCTGAGAAGCTCTAGTAT	AGATACTAGAGCTTCTCAGACATAAAAGCACTCCCTTCCAGCTGCTTG	AAGG>TTTT
m eSR-A		AAAGGTACCCACATAAGAGATGAAAGCTAATG	AAAGCTAGCTAGAATAAAGCTAAGCCAAATCTTG	
REVSEX				
B1	69,533,647-69,535,485	GCGGGTACCTCAGAACTTTGAGAGCCACATT	CGCAGATCTCCTAGCTGGGTTGAGTCAGTTT	
B2	69,534,927-69,536,844	GCGGGTACCAGCCAAGCCATGTTGTTTACTT	CGCGCTAGCGAGACAGGAGAATTGTTTGAACCT	
B3	69,536,414-69,538,223	ATAGGTACCCTCTCAAGTGGCTGGGACTAC	TTTGCTAGCTAAGAAATGACTCCTGCCCTTT	
B4	69,537,713-69,539,563	GCGGGTACCGGAATAGCACTCCATAACAGCTT	CGCAGATCTCTCCCTAGCCATGTGGAAGTAT	
B5	69,539,163-69,540,873	GCGGGTACCTAAGTAGGTGCGTGCTCACTATTAC	CGCGCTAGCCATAGAGACAAACCCATGTTGAGTA	
B6	69,540,389-69,542,207	GCGGGTACCTCTCATCTCCAGTAGAGAATCATTG	CGCGCTAGCGAACTGCTGTTTCCTACATTTCAG	

B7	69,541,752-69,543,558	GCGGCTAGCCTATGGATGTTCACAGTTCCAGTT	CGCAGATCTCAGGACATACTTCCAAGAGGTTATAG	
B8	69,543,081-69,545,033	GCGGGTACCTGCATCTCTATAAGGAAGTACCTGAG	CGCGCTAGCGAGTGAATTCCAGTTACAGAGAAGA	
B9	69,544,553-69,546,686	AAAGGTACCTGTCTGATTCTATCCCATATTCAGG	TTTGCTAGCAATCTTGCTTGAGGACTCAGC	
B10	69,546,373-69,548,414	AAAGGTACCAATGGAGCTCCTCAGATTTCTC	TTTGCTAGCGACCAAGATCTGAATAACAGGAGG	
B11	69,548,178-69,550,323	AAAGGTACCACCTGTAATCCCAGCTACTTGG	TTTGCTAGCTGGAAGATAAGATGAAGCCAGG	
B12	69,550,017-69,552,176	AAAGGTACCCAAGATGCAATGTGGTCTAGTG	TTTGCTAGCCCTAAGAGGAGTGGACAAATGG	
B13	69,551,888-69,554,096	AAAGGTACCCATTAGTGCTCTTCCATCCACTC	TTTGCTAGCCAGAAGTGATCAGTCAATTCAGC	
B14	69,553,867-69,556,031	AAAGGTACCAGAGGAGTCGTCAGGACTCAG	TTTGCTAGCACAGCCCTAAAAGTCTGACTGC	
B15	69,555,753-69,557,834	AAAGGTACCCATACAGGCTTCTATTTCCATATGC	TTTGCTAGCAAGCACAGTGACTCCTCTGTAGC	
B16	69,557,555-69,559,517	AAAGGTACCAAGTCAAATACTGAGTGGCAAGG	TTTGCTAGCCTCAAGTCACTGCTGAGTATCTACC	
eSR-B	69,544,636-69,545,051	ACAGCTAGCAGGGTTGAAAAC*TTTGT*GATGAG	ATACTCGAGTGCAGCATGTTCTGTGAG	
eSR-B ΔSOX9		ATTGAATTCCTTAGAAACAGTTAAGAATTTCCTTTTGGCATTAGTCATTTTG	CAAAATGACTAATGCCAAAAGGAAATTCTTAACTGTTTCTAAGGAATTCAAT	GTT>DEL
m eSR-B		AAAGAGCTCGCTATCTTACCCTTTGGAACTGC	TTTCTCGAGCATCTCTTTGGTCATGAGCAG	
eALDI	70,100,239-70,101,498	ATCTGGTCACATTAATCCTCC	TGATGAGGATGGATTTATGGC	
eALDI ΔSRY		CCAAGAAAAATACAACTCTATCCCTTTGGGGGCATAAGAGGCTGGC	GCCAGCCTCTTATGCCCCCAAAGGGATAGAGTTGTATTTTTCTTGG	ACA>GGG

Supplemental Table 2 Oligos used for sgRNA preparation and PCR genotyping, ALDI and SPR Knock out mouse.

Application	Oligo	Location	Sequence 5'-3' (20-mer sgRNA recognition sequences in bold)
**5' sgRNA preparation**	eSR-B-dist-F	Cas9 cleavage target site is 94 bp 5' to mouse eSR-B	CACC**GGGTGCTAAGACCGAAACAA**
	eSR-B-dist-R		AAACTTGTTTCGGTCTTAGCACCC
	eSR-B-dist-F-T7		TAATACGACTCACTATAGGG**GGGTGCTAAGACCGAAACAA**
**3' sgRNA preparation**	eSR-B-prox-F	Cas9 cleavage target site is 55 bp 3' to mouse eSR-B	CACC**GACCAAAGAGATGCCGTCAT**
	eSR-B-prox-R		AAACATGACGGCATCTCTTTGGTC
	eSR-B-prox-F-T7		TAATACGACTCACTATAGGG**GACCAAAGAGATGCCGTCAT**
**sgRNA preparation**	sgRNA-uni.R	pX330 plasmid	AAAAGCACCGACTCGGTGCC
**Genotyping**	eSR-Bdel-F1	Flanks 5' end of eSR-B	CCAGAGACACCTATAGACAGAGTC
	eSR-Bdel-F2	Internal to mouse eSR-B	ATTAGTCACTTCACATTCAAAGAACCT
	eSR-Bdel-R1	Flanks 3' end of eSR-B	AATAACTGCAAAGGAATGACAC
	Sex-F	X/Y chromosome	GATGATTTGAGTGGAAATGTGAGGTA
	Sex-R	X/Y chromosome	CTTATGTTTATAGGCATGCACCATGTA
**qRT-PCR**	q.Sox9-F	*Sox9*	AGTACCCGCATCTGCACAAC
	q.Sox9-R		TACTTGTAATCGGGGTGGTCT
	q.Wnt4-F	*Wnt4*	CTGGACTCCCTCCCTGTCTTT
	q.Wnt4-R		CATGCCCTTGTCACTGCAA
	q.Foxl2-F	*Foxl2*	AGGGAGAGAATAAAACATTCATGG
	q.Foxl2-R		GCAAACTCCAAGGCCATTAC
	q.Tbp-F	*Tbp*	ACGGACAACTGCGTTGATTTT
	q.Tbp-R		ACTTAGCTGGGAAGCCCAAC
**Application**	**Oligo**	**Location**	**Sequence 5'-3' (18-mer sgRNA recognition sequences in bold)**
**5' sgRNA preparation**	ALDI-dist-F	Cas9 cleavage target site is 122 bp 5' to mouse ALDI element	CACCG**TGTAGGAATTCCATGTAA**
	ALDI-dist-R		AAACTTACATGGAATTCCTACA
	ALDI-dist-F-T7		TAATACGACTCACTATAGGGG**TGTAGGAATTCCATGTAA**
**3' sgRNA preparation**	ALDI-prox-F	Cas9 cleavage target site is 70 bp 3' to mouse ALDI element	CACCG**AGAGTACCCGTGTCAAGA**
	ALDI-prox-R		AAACTCTTGACACGGGTACTCT
	ALDI-prox-F-T7		TAATACGACTCACTATAGGGG**AGAGTACCCGTGTCAAGA**
**sgRNA preparation**	sgRNA-uni.R	pX330 plasmid	AAAAGCACCGACTCGGTGCC
**Genotyping**	ALDI-F1	Flanks 5' end of ALDI	ATGAGAAAGATTGGGGAGGAG
	ALDI-F2	Internal to ALDI	CAACCTTCTCCTTCCTCCA
	ALDI-R1	Flanks 3' end of ALDI	GGTGTGGGTTATCCAAGTATC
	ALDI-R2	Internal to ALDI	CGTCCAAGACTTTACCTTACTG

Sex-F	X/Y chromosome	GATGATTTGAGTGGAAATGTGAGGTA
Sex-R	X/Y chromosome	CTTATGTTTATAGGCATGCACCATGTA

Supplementary Table 3. Oligos used for ChIP-PCR analysis

Target	Primer	Sequence (5'->3')	bp	Tm
eSR-B	eSR-B_1F	CCTTGGCAGCCGAAGATTCAGG	22	57.9
	eSR-B_1R	AGGAAGAGTGCAGCATGTTCTGTGA	25	58.41
eSR-A	eSR-A_1F	AGGCAACAGAACTATTACCCAGG	23	60.05
	eSR-A_1R	GGGAGACCTCTTGTAGGTTACAC	23	59.8
eALDI	ALDI_1F	TGTTTTCTAGGTTTCTTCCGCCT	23	60.18
	ALDI_1R	TTAACTCTCTGAACATCCCCACG	23	60.06
GAPDH	GAPDH_1F	TCGACAGTCAGCCGCATCT	19	60
	GAPDH_1R	CTAGCCTCCCGGGTTTCTCT	20	64
